# Strip-Type Embeddable Shape Sensor Based on Fiber Optics for In Situ Composite Consolidation Monitoring

**DOI:** 10.3390/s22176604

**Published:** 2022-09-01

**Authors:** Shu Minakuchi, Shoma Niwa, Nobuo Takeda

**Affiliations:** 1 Department of Aeronautics and Astronautics, Graduate School of Engineering, The University of Tokyo, 7-3-1 Hongo, Bunkyo-ku, Tokyo 113-8656, Japan; 2 Department of Advanced Energy, Graduate School of Frontier Sciences, The University of Tokyo, 5-1-5 Kashiwanoha, Kashiwa-shi, Chiba 277-8561, Japan

**Keywords:** optical fiber, shape sensing, composite materials, process monitoring

## Abstract

Carbon fibers and resin used in manufacturing carbon fiber-reinforced plastic composite structures flow before the resin solidifies, resulting in disrupted fiber orientation and non-uniform thickness. This process, known as consolidation, is critical for the quality of the composite structure, but no technology exists to measure the deformation in situ. This study proposes a strip-type embeddable shape sensor based on fiber optics for in situ monitoring of consolidation deformation. The sensor consists of a thin, flexible sheet with optical fibers embedded in the upper and lower surfaces of the sheet, and it can monitor out-of-plane bending deformation in composite materials during consolidation. Finite element analysis and experiments are used to evaluate the basic performance of the shape sensor before it is applied to composite gap/lap monitoring. For the first time, the relaxation of consolidation deformation due to the flow of fiber-resin suspension is measured. The proposed sensor will be a powerful tool for elucidating consolidation mechanisms and for validating composite manufacturing simulations.

## 1. Introduction

**Figure 1:**
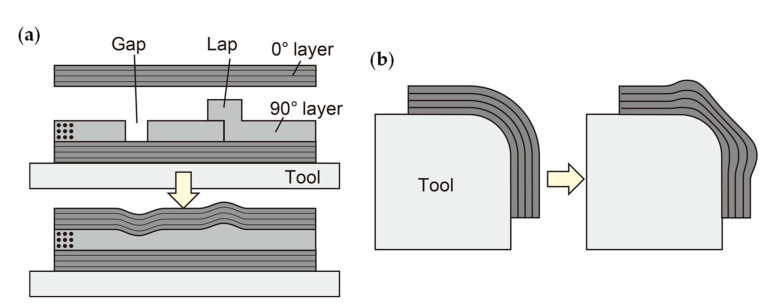
Consolidation of composite laminate with gap/lap (**a**) and composite corner part (**b**).

Carbon fiber-reinforced plastic (CFRP) composites have several applications because of their high mechanical properties and excellent environmental durability. There are various methods for manufacturing CFRP structures, including the method that uses intermediate material sheets called prepreg, in which carbon fibers are impregnated with uncured resin, which can manufacture high-quality CFRP required for aerospace applications [1,2]. Voids created during the heat-curing process of laid-up prepreg sheets significantly degrade the mechanical properties of the cured materials, so the prepreg sheets are cured in an autoclave under high pressure. Specifically, the prepreg sheets laid up on the mold are vacuum bagged and then pressurized in an autoclave from outside the vacuum bag. As the temperature rises, the viscosity of the resin decreases and the voids between the prepreg sheets collapse. Furthermore, the fiber alignment is disrupted in the areas where there are gaps or overlaps between the prepreg sheets or at the corners of the curved areas, and the fiber-resin suspension flows in the areas where there is a pressure gradient (Figure 1). This deformation process, known as “consolidation,” continues until the resin solidifies (i.e., gels) as the curing reaction progresses. The non-uniform thickness and fiber undulation due to consolidation can lead to reduced mechanical properties of the cured composite and high-cost structural assembly [3,4,5,6]. As a result, various experimental evaluations of consolidation deformation have been conducted for many years to clarify the deformation mechanism [7,8,9,10]. Simulation models for predicting consolidation deformation have also been developed, and their validity has been verified by comparison with experiments [11,12,13,14,15,16]. However, most experimental evaluations to date have measured the shape of composite samples after curing and have been unable to accurately evaluate shape changes during consolidation. Recently, in situ measurement using X-ray computed tomography has been conducted [17], but the sample size is limited, and measuring consolidation deformation in a high-pressure environment similar to that of an autoclave is difficult.

For evaluating the cure-induced deformation of composite materials, embedded optical fiber sensors are effective [18,19,20]. Fiber Bragg grating sensors and distributed optical fiber sensors based on Rayleigh and Brillouin scattering have been widely used for evaluation. However, to measure the deformation of a curing composite material with an embedded optical fiber, the optical fiber must be adhered to the composite material by the gelled resin, and the optical fiber and the composite material must deform as one [21,22,23]. In other words, in the consolidation process, where the liquid-state resin flows, the embedded optical fiber slips in the composite material and the deformation cannot be measured. It is necessary to develop a new technique to measure composite deformation even when the optical fiber sensor is not adhered to the composite.

This study proposes an in situ deformation measurement method for composite consolidation using a unique strip-type embeddable shape sensor based on fiber optics. First, an overview of the proposed method is described. Then, basic shape identification tests are conducted to evaluate the performance of the sensor. Finally, the effectiveness of the proposed sensor is demonstrated by monitoring composite gap/lap sections (Figure 1a).

## 2. Strip-Type Embeddable Shape Sensor Based on Fiber Optics

### 2.1. Basic Concept

It is important to note that the 0° layer deformation that occurs during the consolidation process is dominated by out-of-plane bending deformation (Figure 1). The consolidation process can be monitored by embedding a flexible sensor capable of measuring bending deformation and deforming the sensor together with the prepreg. One technique for measuring bending deformation is the use of multicore optical fibers [24,25]. In a standard single-core optical fiber, in which the core is located at the center of the optical fiber, bending deformation does not cause bending strain in the core, whereas in a multicore optical fiber, bending deformation causes strain in the cores located away from the center, corresponding to the distance from the neutral axis and the curvature. Therefore, the curvature distribution can be determined from the strain along the cores, and then, the deformation along the optical fiber can be calculated. Unfortunately, when measuring with a multicore optical fiber, the optical fiber easily twists around its axis, reducing measurement accuracy. This is because the distance of each core from the neutral axis changes as the optical fiber twists, resulting in a large change in the bending strain. Our preliminary experiment embedding a multicore optical fiber in a composite sample confirmed that this torsion-induced measurement instability is a significant problem.

**Figure 2:**
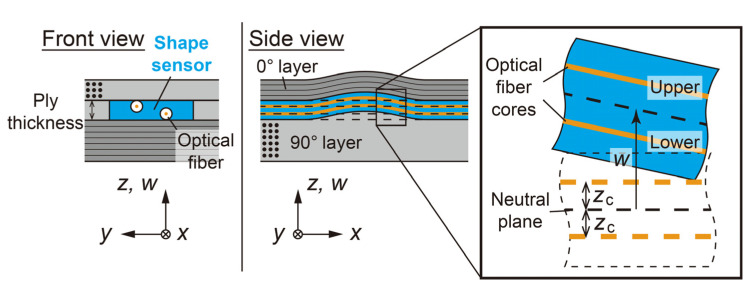
Schematic of strip-type shape sensor embedded in composite.

Therefore, this study proposes a strip-type embeddable shape sensor based on fiber optics (Figure 2). This sensor consists of a thin, flexible sheet and two single-core optical fibers embedded in the upper and lower surfaces of the sheet. When the shape sensor is bent following the deformation of the prepreg, a strain difference is generated in the two optical fibers. This strain difference is measured by a distributed strain measurement system, and the shape is calculated from the strain difference obtained. The sensor is fixed within the layer when embedded because of its stripe shape and is not subject to twisting around the sensor axis. Therefore, bending deformation in the out-of-plane direction can be measured accurately and stably. In this research, one ply of cured prepreg used for CFRP to be monitored is used as a thin, flexible sheet. Therefore, the thickness of the shape sensor is the same as one layer of CFRP, and the sensor can be embedded without changing the thickness of CFRP by removing the prepreg in the sensor embedding area and by inserting the sensor there (Figure 2). Another advantage is that the sensor is made of the same material as the CFRP to be monitored, so the sensor does not become a foreign object when embedded.

The Bernoulli–Euler beam theory is used to calculate the sensor out-of-plane displacement w (Figure 2). First, the curvature κ at each position x is calculated from the strain difference between the two optical fibers on the upper and lower surfaces of the sheet using the following equation.

(1) $$\kappa =\frac{\varepsilon_{\mathrm{lower}}-\varepsilon_{\mathrm{upper}}}{2z_{c}},$$

where $\varepsilon_{\mathrm{upper}}$ and $\varepsilon_{\mathrm{lower}}$ are the strains of the upper and lower optical fibers, respectively, and $z_{c}$ is the distance from the neutral axis to the fiber core. This equation indicates that the bigger the strain differences between the two optical fibers are, the bigger the curvature of the sensor is. Since this study considers small deflections, the following equation holds between the curvature κ and the displacement w.

(2) $$\kappa =\frac{d^{2}w}{dx^{2}}.$$

Therefore, from the distribution of curvature κ obtained from the optical fiber strain measurement using Equation (1), the displacement w of the sensor can be calculated using the following equation.

(3) w=∫κdxdx.

Note that the curvature κ obtained by optical fiber measurement is discrete data at each sampling interval of the measurement system. Therefore, the integral in Equation (3) is calculated by numerical integration according to the midpoint rule.

### 2.2. Measurement System

**Figure 3:**
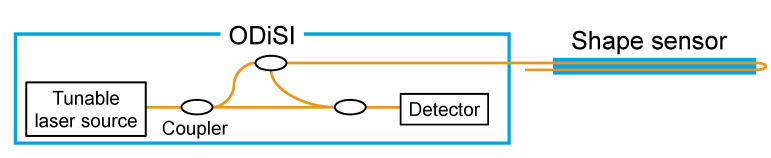
Schematic of measurement system.

This study uses ODiSI A-50 (Luna Innovations, Roanoke, VA, USA), one of the most advanced commercially available instruments, to measure the strain distribution along the optical fiber embedded in the shape sensor (Figure 3). ODiSI uses swept-wavelength interferometry to measure the Rayleigh backscatter as a function of position in the optical fiber [26,27]. The strain is calculated from the amount of shift in the scattered light spectrum. The ODiSI A-50 has a maximum acquisition rate of 2.5 Hz, a minimum sampling interval of 0.4 mm, and a measurement repeatability of ±2 με. Since this study targets micrometer-scale deformation in millimeter-scale areas, ODiSI A-50 with high spatial resolution and measurement repeatability is employed.

### 2.3. Sensor Fabrication

**Figure 4:**
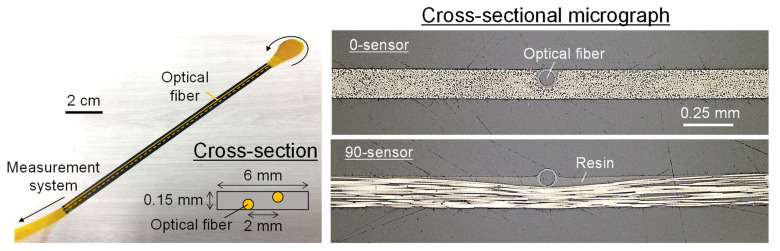
Strip-type shape sensors fabricated using carbon/epoxy prepreg.

Figure 4 shows a shape sensor fabricated using carbon/epoxy prepreg (T700S/2592, Toray, ply thickness 150 μm, glass transition temperature 115 °C). The shape sensor is fabricated as follows. First, single-core optical fibers (polyimide coating diameter 95 μm, Fujikura) are placed horizontally 2 mm apart on the upper and lower surfaces of one ply of the uncured prepreg. Next, the prepreg and optical fibers are sandwiched between metal plates wrapped with 100 μm thick release film and are heated to cure. As a result, the optical fibers are embedded in the thin, flexible sheet of cured CFRP, and bending strain is generated in the optical fiber core when the sheet is bent. Finally, the cured sheet is cut into a 6 mm wide sensor. To evaluate the influence of sensor rigidity on the measurement, two types of sensors were fabricated: a stiff 0-sensor with carbon fibers in the sensor axis direction and a flexible 90-sensor with carbon fibers in the direction orthogonal to the sensor axis. In the 0-sensor, the optical fiber was surrounded by carbon fibers and completely embedded in the sheet, as shown in Figure 4. In contrast, in the 90-sensor, the optical fiber and carbon fibers were orthogonal to each other, so the optical fiber compressed and bent the carbon fibers, and the optical fiber protruded halfway from the sensor surface. The resin overflowing from the compressed carbon fibers generated resin regions on both sides of the optical fiber.

### 2.4. Finite Element Analysis for Sensor Response Evaluation

**Figure 5:**
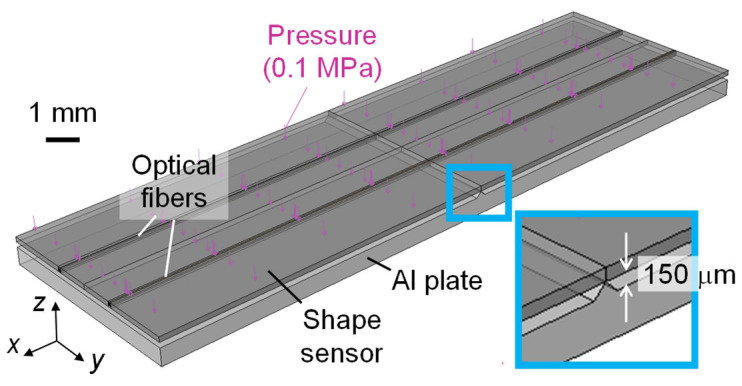
Finite element model for sensor response evaluation.

To evaluate the validity of the proposed shape sensor, a finite element analysis was performed using Abaqus 2021 (Dassault Systemes, Vélizy-Villacoublay, France). Figure 5 shows an overview of the finite element model. The analysis was performed by pressing the shape sensor onto a triangular prismatic protrusion at one atmospheric pressure. The height of the protrusion was 150 μm, which corresponded to the thickness of one ply of the prepreg and was the maximum deformation expected in the demonstration test described below. The shape sensor was modeled based on the cross-sectional observations (Figure 4), and frictionless contact was defined between the sensor and the aluminum plate. Table 1 summarizes the material properties used.

**Figure 6:**
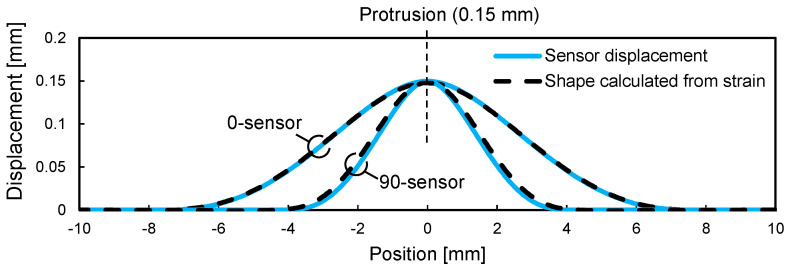
Comparison between sensor displacement and shape calculated from strain distribution along optical fibers.

The distance $z_{c}$ from the neutral axis of the sensor to the optical fiber core was determined to be 25 and 65 μm for the 0-sensor and 90-sensor, respectively, based on the strain distribution in the thickness direction of the shape sensor. Figure 6 shows the sensor shapes calculated using Equations (1) and (3) based on the strain distribution in the optical fiber cores. The sensor displacement obtained from the finite element analysis is also shown for comparison, indicating that the sensor shape could be calculated accurately using the proposed method. To follow the deformation at the height of 150 μm, the 0-sensor and 90-sensor required 6 mm per side and 4 mm per side, respectively. The bending rigidity of the shape sensor is proportional to the elastic modulus of the sheet material (Table 1), and the 0-sensor has a bending rigidity that is more than 10 times that of the 90-sensor. From the analysis results, the 90-sensor with lower rigidity is expected to be more sensitive to prepreg deformation during consolidation.

## 3. Sensor Response Evaluation Test

### 3.1. Materials and Methods

**Figure 7:**
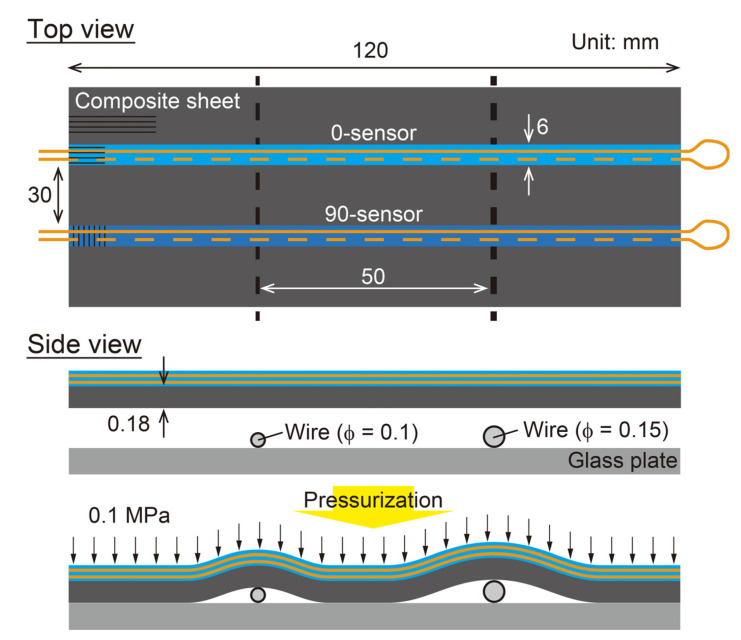
Schematic of experiment setup for sensor response validation.

To measure deformation using the fabricated shape sensors (Figure 4), a demonstration test was conducted. Figure 7 shows a schematic of the test. First, two wires with diameters of 100 and 150 μm were fixed to a glass plate, which was then covered with a stiff composite sheet (T800S/3900-2B, Toray, glass transition temperature 200 °C, *E*_11_ = 180 GPa, thickness 180 μm) and the two shape sensors. Convex shapes of two different heights were generated on the shape sensors by vacuum bagging the entire specimen. Note that the composite sheet was used to prevent the sensor from breaking due to local deformation where the sensor and wires came in contact. The curvature was calculated from the strain measured by a distributed strain measurement system (ODiSI A-50) using Equation (1), and the deformation was calculated using Equation (3). The sampling interval for strain measurement was set to 1 mm for accurately and stably measuring changes in the strain distribution caused by deformation. Additionally, to evaluate the stability of the sensor response in the heating environment expected during composite curing, a test was conducted, in which the vacuum bagged specimen was heated to 100 °C in an oven.

### 3.2. Results

**Figure 8:**
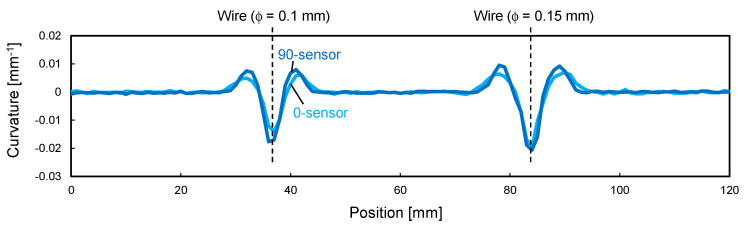
Curvature distribution calculated from strain data.

**Figure 9:**
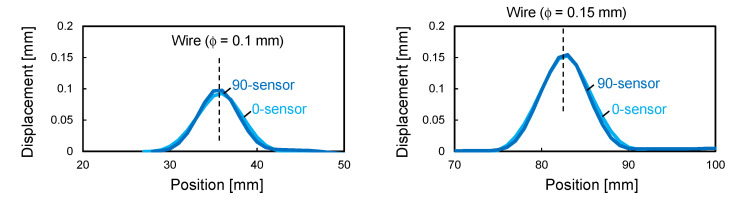
Sensor shape calculated from curvature.

**Figure 10:**
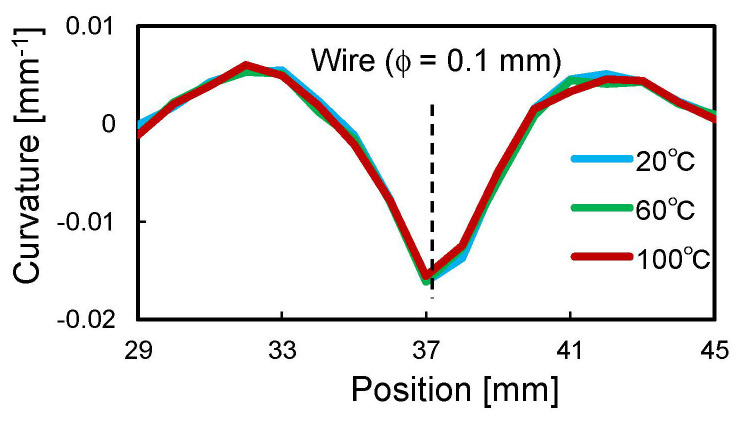
Curvature distribution of 0-sensor at 100 μm wire position depending on temperature.

Figure 8 shows the curvature distribution measured by the two sensors. The M-shaped curvature distribution caused by the convex deformation was successfully measured, and the curvature magnitude was greater in the higher convex part (i.e., 150 μm wire). The repeatability of strain measurement by ODiSI A-50 was ±2 με, and from Equation (1), the measurement error of curvature was calculated to be ±8 × 10^−5^ mm^−1^ for the 0-sensor and ±3 × 10^−5^ mm^−1^ for the 90-sensor, respectively. The micrometer-scale deformation caused sufficiently large curvature changes compared to the measurement error, confirming that the proposed sensor can sensitively detect micrometer-scale deformations. It is interesting to note that the 0-sensor had a slightly wider and smaller curvature than the 90-sensor, especially at the 100 μm wire position. This was because the bending rigidity of the 0-sensor was very high, which prevented the sensor from completely following the steep deformation of the composite sheet. Figure 9 shows the sensor shape calculated from this curvature distribution using Equation (3). The convex shapes with heights of 100 and 150 μm were obtained with good accuracy, confirming that the fabricated shape sensor can be used to quantitatively evaluate micrometer-scale deformation. In the heating test using an oven, the curvature of the sensor did not change as the temperature increased. As an example, Figure 10 shows the curvature distribution of the 0-sensor at the 100 µm wire position. Thus, it was confirmed that stable shape evaluation is achievable, even during composite curing under heating conditions. In the next section, this sensor system is applied to the consolidation monitoring of a composite gap/lap region.

## 4. Consolidation Monitoring of Composite Gap/Lap

### 4.1. Materials and Methods

Recently, large-scaled composite structures have been manufactured using an automated lay-up machine that aligns and laminates several narrow prepreg sheets known as slit tapes. However, gaps and overlaps occur between the slit tapes due to the limit of lay-up accuracy. These gaps and overlaps cause changes in fiber orientation and resin-rich areas during consolidation (Figure 1a), resulting in shape loss and mechanical property degradation [3,4]. This consolidation deformation is critical for the quality of the cured composite, but it has never been observed in situ. Therefore, the authors conducted a monitoring experiment of a gap/lap section using the proposed shape sensor.

**Figure 11:**
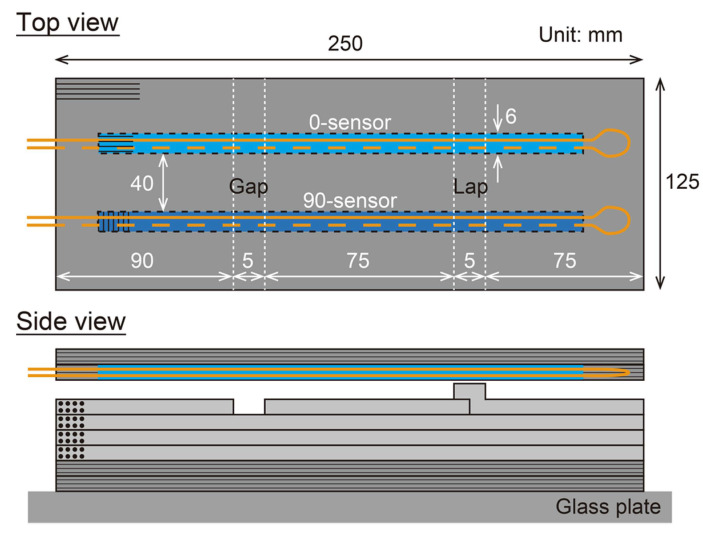
Schematic of specimen for consolidation monitoring.

Figure 11 shows a schematic of the specimen. The carbon/epoxy prepreg (T700S/2592, Toray, ply thickness 150 μm) was used as the material, and the stacking sequence was [0_2_/90_2_]_S_. The prepreg sheets were laid up by hand, with a 5 mm wide gap and lap on one ply of the 90° layer just below the upper 0° layer. Two 6 mm wide sections of one ply of 0° prepreg directly above the gap/lap were removed, and the two kinds of 6 mm wide shape sensors were inserted there. The entire specimen was vacuum bagged and then cured in an autoclave via heating. The curing process was as follows. Following vacuuming, the autoclave was pressurized to 0.3 MPa at 20 °C, then heated to 100 °C at 2 °C/min, maintained at 100 °C for 90 min, and then cooled to room temperature at 2 °C/min. The curvature of the shape sensor during this curing process was measured by the measurement system (ODiSI A-50). The measurements were taken manually when predetermined temperatures were reached, and each measurement took less than 5 s to complete. After curing, the thickness distribution of the specimen was measured using a 3D scanner (ATOS Core Essential Line 5M, GOM Metrology, Braunschweig, Germany), and the cross section of the specimen was polished for observation.

**Figure 12:**
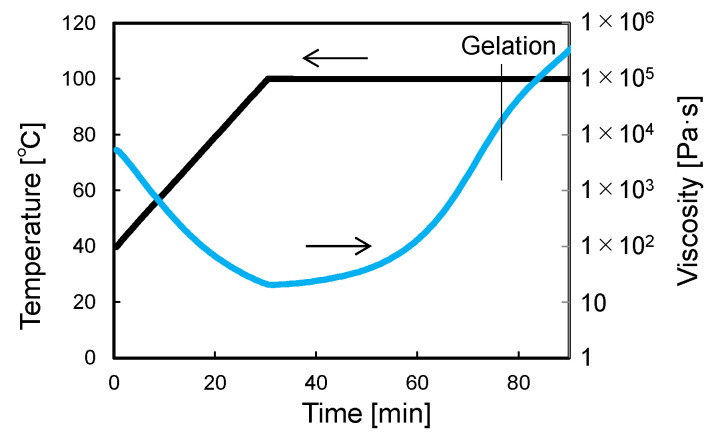
Viscosity of epoxy resin squeezed from T700S/2592 prepreg.

Additionally, as a preliminary preparation, the viscosity of the epoxy resin during the curing process was evaluated using a rheometer (MCR102, Anton Parr, Graz, Austria). The resin was squeezed out of the T700S/2592 prepreg. Figure 12 shows the measured changes in viscosity. The viscosity decreased with increasing temperature but increased after reaching the holding temperature of 100 °C. At 100 °C, the viscosity increased slightly at first and then increased rapidly after 30 min. Based on the time at which the storage modulus of the curing resin exceeded the loss modulus, the resin solidification (i.e., gelation) was identified as occurring 45 min after 100 °C was reached.

### 4.2. Results and Discussion

**Figure 13:**
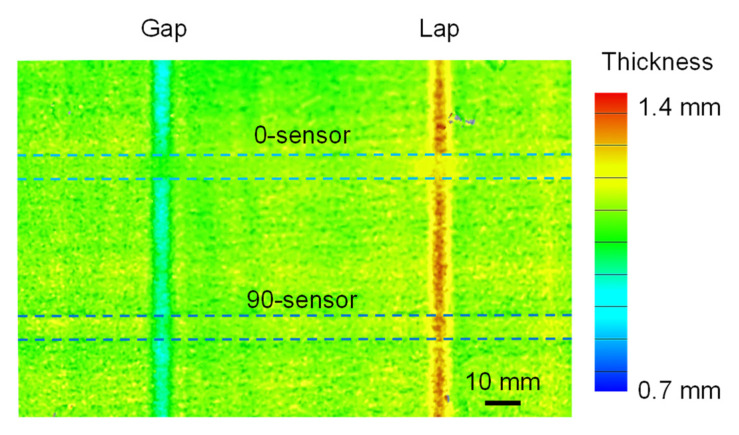
Thickness distribution around gap/lap.

Figure 13 shows the measured 3D shape of the specimen after curing. Concave deformation due to the prepreg gap and convex deformation due to the prepreg lap were induced. During prepreg lay-up, both gap and lap were introduced into the specimen at a width of 5 mm, but the deformation occurred in wider areas. It is important to note that in the 90-sensor area, the deformation was equivalent to that in the no-sensor area, whereas in the 0-sensor area, the concavo-convex shape caused by the prepreg gap/lap was smoothed out. This was because the 0-sensor was too rigid, which disturbed the prepreg deformation during consolidation. As mentioned above, the bending rigidity of the 0-sensor was more than 10 times that of the 90-sensor. This result indicates that a shape sensor with low bending rigidity should be used to measure deformation during consolidation. Therefore, the response of the 90-sensor is discussed below.

**Figure 14:**
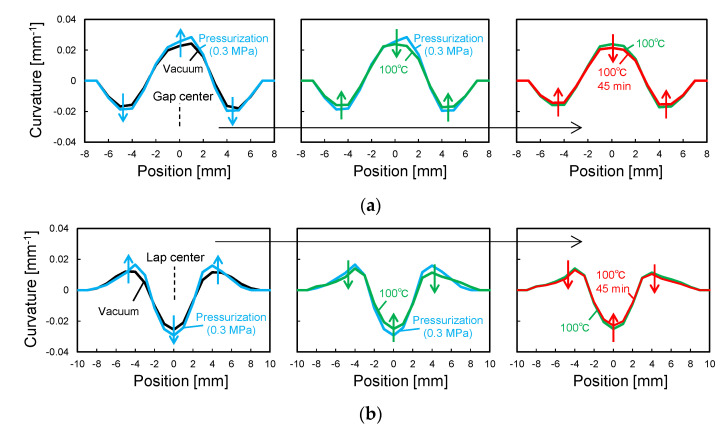
Curvature change of 90-sensor during consolidation. (**a**) Gap. (**b**) Lap.

**Figure 15:**
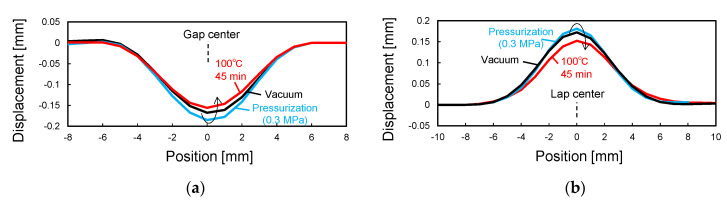
Shape calculated from curvature distribution. (**a**) Gap. (**b**) Lap.

**Figure 16:**
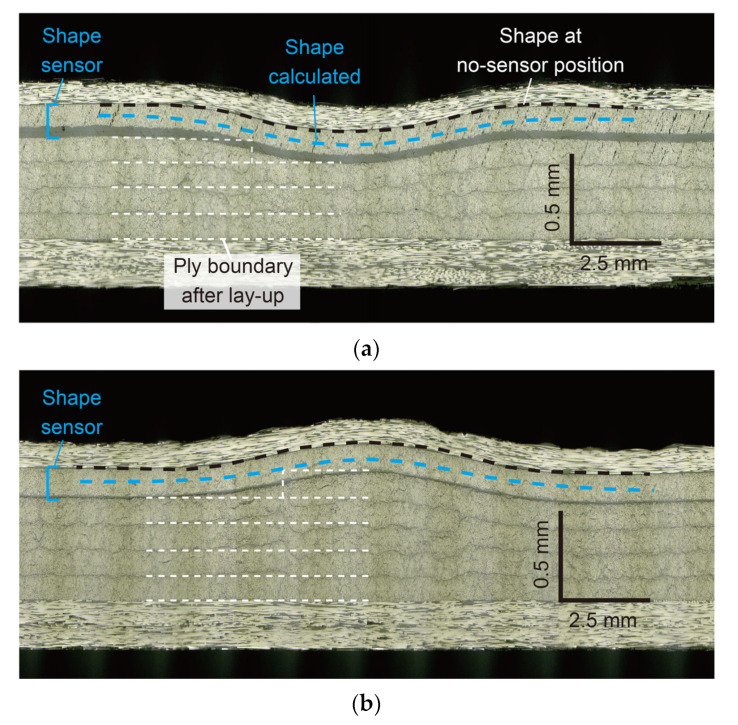
Cross-sectional micrographs of the specimen after curing. Note that the photos were compressed horizontally by a factor of five to make deformations more clearly visible. (**a**) Gap. (**b**) Lap.

Figure 14 shows the curvature change in the 90-sensor during the consolidation process. Figure 15 shows the shape calculated from this curvature distribution. By vacuum bagging the laid-up prepreg specimen, the shape sensor was deformed together with the surrounding 0° prepreg into the concavo-convex shape along the gap and lap, and W- and M-shaped curvature distributions were measured. Subsequently, as the pressure in the autoclave was increased to 0.3 MPa, the concavo-convex shape deepened, increasing the curvature. As the temperature increased, the curvature increased slightly up to 50 °C and then decreased. The decrease in curvature with heating is due to the decrease in the resin viscosity (Figure 12). The fiber and resin flowed, resulting in a shallower concavo-convex shape. Even after reaching the holding temperature of 100 °C, the curvature continued to decrease slightly, but the change stopped 45 min after reaching 100 °C when the resin gelled. Figure 16 shows cross-sectional micrographs of the gap/lap after curing. To make the deformation more visible, the micrographs were compressed horizontally by a factor of 5. First, the overall deformation induced was the same as in the area where the sensor was not embedded (i.e., no-sensor position, black dashed line), confirming that the consolidation deformation was not disturbed by the sensor embedding. Furthermore, the shape obtained by the sensor (blue dashed line) and the deformation at the gap/lap section agreed well. On the left side of the cross-sectional micrographs, the white dashed line indicates the ply boundary of 90° prepreg at the time of lay-up. On the right side, the dark resin-rich line indicates the ply boundary of the 90° layer after curing. The portions of the 90° prepreg that were protruding at the gap/lap edges were pushed into the 90° layer by the bent 0° layer during consolidation, and the cavity between the 0° and 90° prepreg was filled with flowing fibers and resin. The change in curvature and shape of the sensor suggests that the consolidation process proceeded as follows. When vacuumed and pressurized at 20 °C, the 0° layer was pressed against the laid up 90° layer with gap/lap, but the 90° layer did not flow and preserved some of its initial shape due to the high resin viscosity. As a result, the 0° layer was in partial contact with the 90° layer, resulting in steep deformation of the 0° layer. However, as the resin viscosity decreased with increasing temperature, the high pressure generated at the contact regions caused the fiber/resin suspension to flow in the 90° layer, which relaxed the deformation of the 0° layer to reach the final state. This flow-induced deformation relaxation has also been reported in gap-filling simulations [15,16] but has yet to be experimentally verified. The shape sensor proposed in this study enables in situ observation of such previously uncapturable consolidation deformation, and it will be a powerful tool for elucidating deformation mechanisms and for validating simulations.

## 5. Conclusions

This study proposed a strip-type embeddable shape sensor based on fiber optics that enables in situ monitoring of composite consolidation deformation. The sensor consists of a thin, flexible sheet with optical fibers embedded in the upper and lower surfaces of the sheet. The basic response performance of the shape sensor was first evaluated using finite element analysis and experiments. The results confirmed that the sensor with low bending rigidity is preferred. The sensor was then applied to composite gap/lap monitoring. For the first time, the relaxation of consolidation deformation due to the flow of fiber/resin suspension was measured. The shape obtained by the sensor and the deformation at the gap/lap section agreed well, confirming the validity of the proposed sensor system. The proposed sensor will be a powerful tool for elucidating consolidation deformation mechanisms and for validating composite manufacturing simulations.

## Figures and Tables

**Table 1:** Material properties used for finite element analysis.

	T700S/2592 Unidirectional	Cladding and Core	Polyimide Coating	Aluminum
Elastic moduli (GPa)				
*E*_11_	135	73.1	1.5	70
*E*_22_	8.5			
*G*_12_	4.8			
*G*_23_	2.7			
Poisson’s ratios				
ν_12_	0.34	0.16	0.25	0.35
ν_23_	0.49			
